# Determination and Ecological Risk Assessment of Organophosphate Esters in Drinking and Environmental Waters by Automated Liquid–Liquid Extraction Coupled with GC-MS/MS

**DOI:** 10.3390/molecules31122131

**Published:** 2026-06-17

**Authors:** Guowei Wang, Hongmei Hu, Yanjian Jin, Tiejun Li, Zhenhua Li, Yunyong She, Qinglin Mu, Yuanming Guo

**Affiliations:** 1 Institute of Marine and Fisheries, Zhejiang Ocean University, Zhoushan 316021, China; 2 Key Laboratory of Sustainable Utilization of Technology Research for Fisheries Resources of Zhejiang Province, Zhejiang Marine Fisheries Research Institute, Zhoushan 316021, China; 3 Marine Ecological and Environmental Monitoring Center of Zhejiang Province, Zhoushan 316021, China

**Keywords:** organophosphate esters, automated liquid–liquid extraction, gas chromatography–tandem mass spectrometry, ecological risk, water

## Abstract

Organophosphate esters (OPEs) are ubiquitous in the global water environment and may pose potential risks to aquatic ecosystems and human health. Herein, we present a simple and efficient method for accurate quantification of nineteen OPEs in water based on automated liquid–liquid extraction (LLE) with dichloromethane and hexane, followed by gas chromatography–tandem mass spectrometry (GC-MS/MS) with isotope dilution calibration. This method demonstrated a negligible matrix effect, satisfactory recoveries (70–120%), and low limits of detection (0.46–2.32 ng/L). A total of 3, 9, 10, and 11 OPEs were detected in Wahaha pure water, tap water, river water, and seawater, respectively, with total OPE (∑OPE) concentration ranges of 8.23–18.5 ng/L, 159–218 ng/L, 202–623 ng/L, and 111–175 ng/L, respectively. Among the detected OPEs, tris(2-chloroethyl) phosphate (TCEP) and tris(1-chloro-2-propyl) phosphate (TCIPP) were the predominant compounds in four test water matrices. The risk quotient (RQ) results revealed that OPEs exhibited a low risk to algae, crustaceans and fish in the river water of Zhoushan and seawater of Sanmen Bay. Overall, the proposed method is sensitive and reliable for routine OPE analysis in drinking and environmental waters.

## 1. Introduction

Organophosphate esters (OPEs) are synthetic phosphate derivatives substituted with alkyl, halogenated alkyl, and aryl groups [1]. They have been widely used as flame retardants, plasticizers, and defoamers in agriculture, medicine, and industrial fields [2]. Due to restrictions on certain brominated flame retardants (hexabromocyclododecane and polybrominated diphenyl ethers (PBDEs)), OPEs have emerged as the predominant flame retardants in recent years [3]. However, studies have increasingly confirmed that numerous negative effects on health are associated with exposure to OPEs [4]. The United States, Canada, and the European Union have banned the use of tris(2-chloroethyl) phosphate (TCEP), tris(1,3-dichloro-2-propyl) phosphate (TDCPP), and tris(1-chloro-2-propyl) phosphate (TCIPP) in commercial products, whereas China listed TCEP and isopropylphenyl phosphate (IPPP) in its Priority Control Chemicals Inventory in 2020 [5]. OPEs are commonly incorporated into materials through physical mixing rather than chemical bonding and can thus be easily released into the environment through leaching, abrasion, and volatilization [6]. Ubiquitous OPEs in air, dust, soil, sediment, water, foodstuffs, organisms, and even human biological samples are globally well documented [4,7]. For instance, several studies have demonstrated that OPEs occur in various water bodies, including surface water, groundwater, drinking water, seawater, wastewater, and rainwater, at concentrations ranging from ng/L to μg/L [1,8,9,10,11]. Therefore, efficient and reliable analytical methods are urgently required to quantify OPEs in water environments and evaluate their potential health and ecological risks. Current analytical protocols for aqueous OPEs are mostly matrix-specific, and comprehensive methods covering both drinking water and environmental water (i.e., pure water, tap water, river water, seawater) are still lacking, which is a prominent research gap in this field.

Currently, the most commonly used methods for OPEs include gas chromatography [12,13], gas chromatography–mass spectrometry (GC-MS) [1,14,15,16], gas chromatography–tandem mass spectrometry (GC-MS/MS) [10,17,18,19,20,21,22,23,24], high-performance liquid chromatography–tandem mass spectrometry (HPLC-MS/MS) [2,6,11,25,26,27], ultra-performance liquid chromatography–tandem mass spectrometry (UPLC-MS/MS) [4,8,28,29,30,31,32,33,34,35,36], and ultrahigh-performance liquid chromatography–Q Exactive hybrid quadrupole-Orbitrap high-resolution mass spectrometry (UPLC-Q-Orbitrap HRMS) [37]. LC-MS/MS exhibited excellent sensitivity and specificity, demonstrating suitability for analyzing both volatile and non-volatile OPEs; however, this analytical technique suffered significant susceptibility to matrix effects [28]. When analyzing complex matrices, GC-MS/MS showed superior selectivity, sensitivity and accuracy relative to GC-MS [38], making it the method of choice for analyzing volatile OPEs. Nevertheless, these methods only involved limited OPEs, typically no more than 13 [1,12,15,18,19,20,21,25,29,31]. This lack of data might possibly originate from difficulties with OPE extraction from a complex matrix or limit of detection (LOD) incompatibility with environmental levels. Before instrumental analysis, trace OPEs in water samples require preconcentration, which can be achieved via methods such as liquid–liquid extraction (LLE) [14,17,21,22,29,37], solid-phase extraction (SPE) [1,4,10,11,18,19,20,24,26,27,30,31,32,33,34,35], dispersive solid-phase extraction (dSPE) [15], dispersive liquid–liquid microextraction (DLLME) [36], hollow-fiber liquid-phase microextraction (HF-LPME) [16], solid-phase microextraction (SPME) [13], headspace SPME [12], and magnetic solid-phase extraction (MSPE) [2,8]. Among them, dSPE features low cost and high throughput but weak enrichment capability. DLLME achieves efficient enrichment with low solvent consumption, while its performance is easily affected by emulsification and high salinity. HF-LPME has strong anti-interference ability yet low analysis efficiency. Solvent-free SPME is convenient for automation and field testing, but it suffers from low recoveries for weakly polar and low-volatility OPEs. MSPE enables rapid separation and selective extraction, but material agglomeration limits its practical use in complex water matrices. Normally, LLE and SPE were the most used techniques for OPE extraction from water environments due to their high sensitivity and robustness. However, SPE features tedious operations, low throughput and relatively high solvent usage. Plastic cartridges cause background contamination, while matrix effects, carryover and column clogging are also common issues [1]. In contrast, carryover and memory effects are largely absent in LLE, given that each sample is processed with a newly added solvent. Nevertheless, conventional LLE still has several inherent flaws. To address these issues, various modified and novel LLE methods have been developed to shorten extraction time, boost extraction efficiency and cut down organic solvent consumption.

The structural diversity of OPEs results in a wide range of chemical properties, ranging from very polar to highly hydrophobic. The goal of this study is to develop a simple and robust automated LLE coupled with stable isotope dilution GC-MS/MS for quantifying 19 OPEs (0.80 < log Kow < 9.49) in drinking and environmental water samples. Matrix effects were eliminated using nine isotope-labeled internal standards (ILISs). No plasticware was used throughout the automated LLE-GC-MS/MS procedure, which greatly reduces system blank contamination. The molecular structure and physicochemical properties of target OPEs are displayed in Supplementary Materials, Figure S1 and Table S1, including eight alkyl OPEs, four Cl-OPEs, and seven aryl OPEs. After optimization (e.g., extraction solvent) and validation, the proposed method was applied to detect OPEs in various real water samples including Wahaha pure water, tap water, river water from Zhoushan, and seawater from Sanmen Bay, East China. Then, the occurrence and ecological risks in the survey area were also discussed and assessed, providing scientific methods and a basis for the pollution control and risk management of organophosphorus flame retardant.

## 2. Results and Discussion

### 2.1. Optimization of Automated Liquid–Liquid Extraction

**Figure 1:**
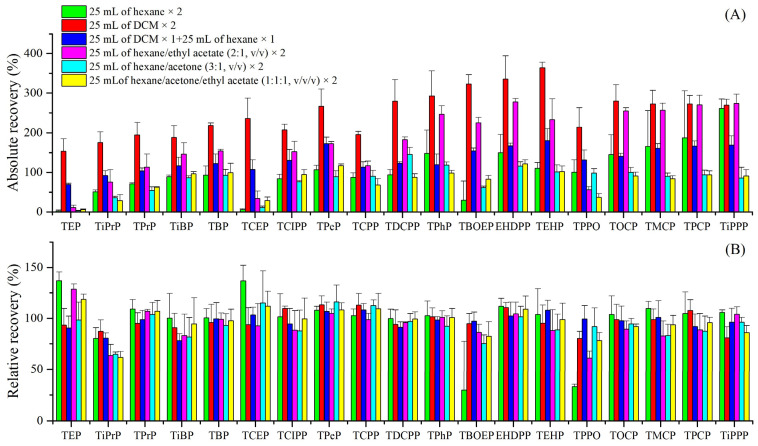
Effect of extraction solvents on absolute recovery (**A**) and relative recovery (**B**) (*n* = 3).

Previous studies have applied LLE for the extraction of OPEs in water [14,17,21,22,29,37]. To address the limitations of conventional manual LLE, which is both time- and labor-intensive, this study employs mechanical agitation for automated LLE, thereby enhancing experimental efficiency [39]. Considering the large differences in the polarities and volatilities of target analytes (log*K*_OW_ ranging from 0.80 to 9.49, water solubility ranging from 2.59 × 10^−5^ to 5.0 × 10^5^ mg/L, vapor pressure ranging from 2.6 × 10^−9^ to 0.39 mm Hg, Table S1), extraction solvent selection is critical, and dichloromethane (DCM) was the most commonly used in previous work [14,17,21,22,29,37]. Six solvents were assessed for their efficiency in extracting spiked ultrapure water (spiked at 100 ng/L) by external standard method (absolute recovery). As shown in Figure 1A, DCM exhibited the highest extraction efficiencies for 19 OPEs, with absolute recoveries in the range of 154–364%, followed by DCM and hexane (69–180%). However, when the other four extraction solvents were utilized, the absolute recoveries of three to four OFEs were less than 60%. For instance, using hexane/acetone (3:1, *v*/*v*), the absolute recoveries of TEP, TiPrP, TPrP, and TCEP were 3%, 36%, 54%, and 11%, respectively. The high recoveries (154–364%) of DCM are mainly attributed to matrix enhancement from co-extracted substances. Furthermore, DCM also exhibits the highest extraction capacity for OPEs of different polarity with little analyte loss. Other extract solvents have low affinity for polar OPEs, leading to low recoveries.

The above recovery results indicated a severe matrix effect for OPE quantification in water using external standard calibration. As such, nine isotopically enriched OPEs were utilized for recovery estimation (relative recovery in this mode) using isotope-labeled internal standard (ILIS) calibration (which will be discussed in Section 2.2). As illustrated in Figure 1B, a water sample extracted with 25 mL of DCM twice, or once with 25 mL of DCM and once with another 25 mL of hexane, offered superior relative recoveries in the ranges of 80–113% (mean 98%) and 78–108% (mean 97%), respectively. This demonstrated that the ILISs with identical or similar molecular structures and physicochemical properties to the target OPEs were capable of correcting the matrix interference from extraction [20]. Nevertheless, the relative recoveries of one to four OPEs (namely TEP, TiPrP, TCEP, TBOEP, and TPPO) were less than 65% or more than 125% by using other four extraction solvents. Considering the recoveries and toxicity of both, DCM and hexane were each selected once as the extraction solvent for automated LLE.

### 2.2. Matrix Effect

**Figure 2:**
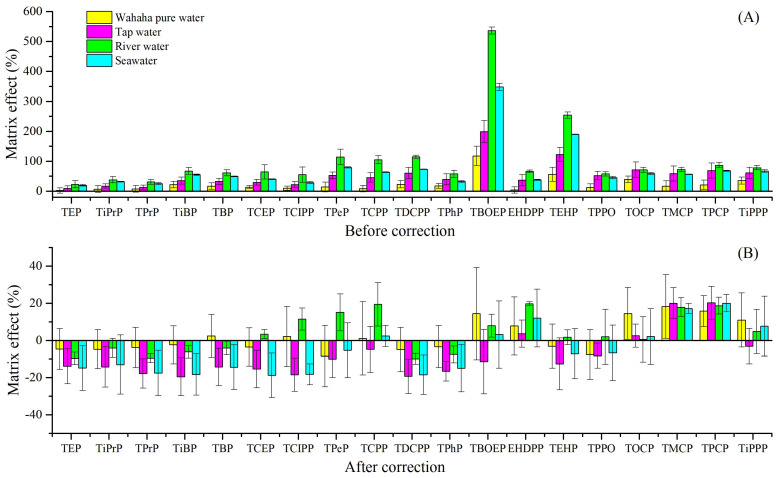
Matrix effects in four different water matrices before (**A**) and after (**B**) correction.

GC-MS/MS has become an essential analytical tool due to its exceptional sensitivity and selectivity. However, the unavoidable matrix effects (MEs), which may suppress or enhance analyte signals, frequently compromise quantification accuracy in GC-MS/MS analyses [40]. Matrix effects were calculated by comparing the peak areas of target OPEs in the matrix and in the standard solution according to the following equation, ME = ((A_m_ − A_0_)/A_s_ − 1) × 100%, where A_m_ and A_s_ are the peak areas of target OPEs in the spiked matrix and standard solution at the same concentration, respectively, while A_0_ is the background signal in the unspiked matrix [41]. The ME values were categorized into negligible (≤±20%), minor (>±20% and ≤±50%), and significant (>±50%) [42]. Figure 2A illustrates varying degrees of signal enhancement in Wahaha pure water (ME range 2–117%, mean 23%), tap water (9–199%, 54%), river water (23–536%, 103%), and seawater (20–349%, 72%). For instance, among the 19 OPEs in seawater, up to 10 OPEs suffered from major matrix effects (55–349%), while 8 OPEs suffered from minor matrix effects (25–49%), and only TEP suffered from negligible matrix effects (20%). Since ILIS is widely recommended to compensate for matrix effects and improve quantification accuracy [20], nine ILISs were employed for this purpose. After calibration with ILIS, the matrix effects became negligible (ME values within ±20%, Figure 2B). Therefore, the internal standard method is recommended for quantification of OPEs in water. It should be critically noted that matrix effects reached as high as 536% for TBOEP in river water before ILIS correction, which demonstrates that the GC-MS/MS instrumental system alone has weak inherent robustness and is extremely susceptible to signal enhancement interference induced by co-extracted impurities in water extracts. If quantified without internal standard calibration, severe overestimation would occur for all target OPEs.

### 2.3. Evaluation of the Method’s Performance

The linearity, limit of detection (LOD, S/N = 3), limit of quantitation (LOQ, S/N = 10), and precision of the developed automated LLE-GC-MS/MS method were validated under the optimum conditions. As displayed in Table 1, good linearity with correlation coefficients of 0.9905–0.9986 was achieved when the 19 target OPEs’ concentrations ranged from 5 to 1000 μg/L. The instrumental LODs and LOQs were in the ranges of 1.15–5.80 μg/L and 3.55–19.6 μg/L, respectively. By considering sample pretreatment, the method’s LODs and LOQs were in the ranges of 0.46–2.32 ng/L and 1.42–7.83 ng/L, respectively. Intra-day (*n* = 6) and inter-day (*n* = 6) precision at 100 ng/L show relative standard deviations (RSDs) below 20% and 18%, respectively, demonstrating good precision. A typical chromatogram of the 19 OPEs’ standard mixture solution is shown in Figure S2, and all target compounds achieved good chromatographic separation. Considering no available certified reference materials for OPEs in water matrices, the method’s accuracy was validated by spiked recovery experiments at four spike levels (20, 50, 100, and 400 ng/L) using four real water matrices. As listed in Table S2, the spike recoveries are in the ranges of 71–96% with 6.4–17.7% RSDs (Wahaha pure water), 70–95% with 6.5–18.0% RSDs (tap water), 70–87% with 5.8–16.9% RSDs (river water), and 70–120% with 1.2–17.7% RSDs (seawater), demonstrating satisfactory accuracy and acceptable precision.

In comparison with previous methods (Table 2), the proposed plastic-free automated LLE-GC-MS/MS method achieved satisfactory accuracy and precision and offered comparable method LODs to SPE-UPLC-MS/MS (LODs: 0.01–1.72 ng/L) [31], SPE-GC-MS/MS (0.03–0.25 ng/L) [19], automated LLE-GC-MS (0.68–2.96 ng/L) [21], and automated LLE-UPLC-MS/MS (0.06–1.57 ng/L) [29] but superior to those obtained with HS-SPME-GC-NPD (1.4–135.6 ng/L) [12], DIA-LC-MS/MS (1.5–30 ng/L) [25], dSPE-GC-MS (53–98 ng/L) [15], automated LLE-GC-MS (0.4–12 ng/L) [18], SPE-GC-MS/MS (0.3–24 ng/L) [20], and SPE-GC-MS (0.3–12.3 ng/L) [1]. In addition, the developed method has advantages including negligible background contamination, simplicity, rapidness, efficiency, low cost, matrix robustness, reduced organic solvents, and wide OPE coverage. Therefore, the developed method is reliable and practical for quantifying OPEs in environmental water samples.

### 2.4. Real Water Analysis

**Figure 3:**
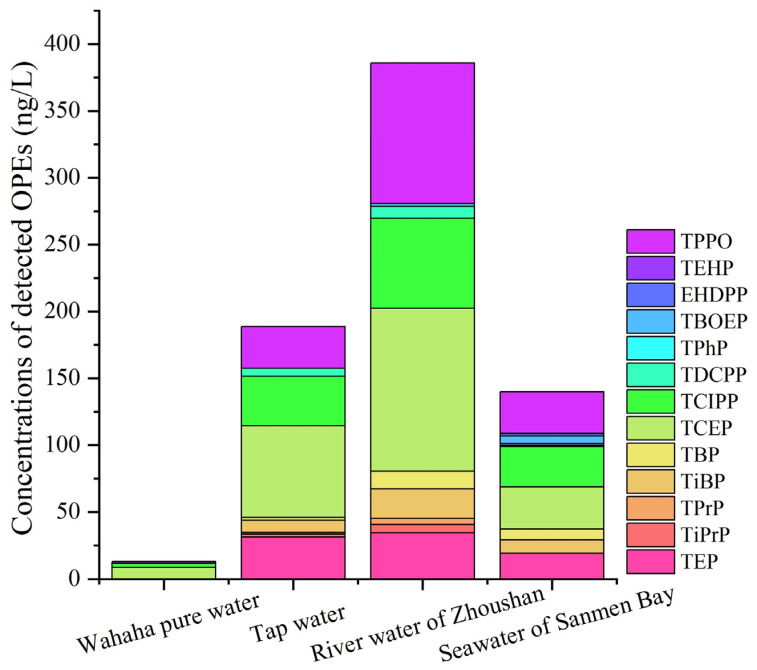
Concentrations of detected OPEs in four types of water.

Twenty water samples representing four distinct water types were analyzed by the developed automated LLE-GC-MS/MS method to verify its applicability, with results shown in Supplementary Materials, Table S3 and Figure 3. A total of 3, 9, 10, and 11 OPEs were detected in Wahaha pure water, tap water, river water, and seawater, respectively, with total OPE (∑OPE) concentration ranges of 8.23–18.5 ng/L, 159–218 ng/L, 202–623 ng/L, and 111–175 ng/L, respectively. The mean concentration of ΣOPEs in river water (386 ng/L) was higher than those found in tap water (189 ng/L), seawater (140 ng/L), and Wahaha pure water (12.9 ng/L). Lower concentrations of OPEs found in seawater can be explained by the dilution effect [26]. In China, people commonly drink boiled tap water; however, the median ∑OPEs slightly increased from 192 ng/L to 212 ng/L after boiling [32]. The potential health risks associated with a high level of OPEs in tap water should be taken seriously. Installing home water filters is recommended to decrease the concentration of OPEs in tap water. On the other hand, although the concentrations of OPEs in Wahaha pure water were much lower than those in tap water, the bottle-sealing process or the use of recycled PET bottles may increase the risk of OPE contamination.

The detection frequencies of TPPO in Wahaha pure water, TBOEP in river water, and TDCPP, TPhP, TBOEP, EHDPP, and TEHP in seawater were 33.3%, 33.3%, and 12.5–50%, respectively, while those of other detected OPEs were all 100%. Regarding composition, Wahaha pure water was dominated by TCEP and TCIPP, tap water was dominated by TCEP, TCIPP, TPPO, and TEP, and river water and seawater were dominated by TCEP, TPPO, and TCIPP. The mass fraction of Cl-OPEs (TCEP and TCIPP) accounted for 44–91% of ∑OPEs in four water matrices, which is related to their extensive use in household products, high hydrophilicity, strong environmental persistence and difficult degradation [31]. This specific chemical pattern was compatible with the results found in bottled drinking water from South Korea [1], barreled water from Eastern China [32], tap water distributed in Wuhan, central China [33], surface water from the Eastern Route of the South-to-North Water Diversion Project, China [27], and seawater from the Changjiang Estuary to the adjacent East China Sea [19]. Furthermore, TPPO, which is widely used as an intermediate and catalyst in organic synthesis and pharmaceutical products [23], was also one of the most prevalent OPE congeners in seawater from Xiangshan Bay, East China Sea [10] and river water from Yarlung Tsangpo River and its main tributaries on the Tibetan Plateau [22]. Considering the extensive global usage of OPEs and their potential toxicity, it is of great significance for the systematic assessment of OPEs in various drinking and environmental waters.

### 2.5. Ecological Risk Assessment

There is growing scientific concern about the effects of OPEs on ecosystems due to their persistent release and potential toxicity. In this study, the risk quotient (RQ) was employed to assess the ecological risks posed by OPEs in the river water of Zhoushan and seawater of Sanmen Bay. The RQ is defined as the ratio of the measured environmental concentration to the predicted no-effect concentration (PNEC) [22]. The PNEC values were calculated by dividing the acute toxicity data by an assessment factor (AF) of 1000 [22]. The RQ values were categorized into low risk (RQ ≤ 0.1), medium risk (0.1 < RQ ≤ 1), and high risk (RQ > 1). The sum of each individual RQ value (ΣRQ) represents the combined contamination risk of the total OPEs [22].

**Figure 4:**
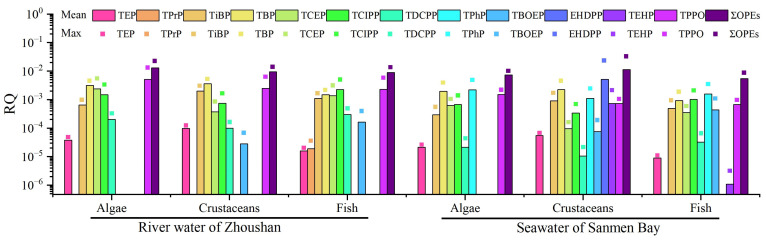
RQ values of OPEs detected in aquatic organisms at different trophic levels.

With the exception of TiPrP (which lacks toxicity data), the PNECs for the other 12 detected OPEs are provided in Supplementary Materials, Table S4, while the calculated RQ values for the trophic levels of the three representative organisms (algae, crustaceans, and fish) are illustrated in Figure 4. The results show that the RQs of TEP, TPrP, TiBP, TBP, TCEP, TCIPP, TDCPP, TBOEP, TPPO and ΣOPEs in river water, as well as TEP, TiBP, TBP, TCEP, TCIPP, TDCPP, TPhP, TBOEP, EHDPP, TEHP, TPPO and ΣOPEs in seawater, for all three trophic levels were far below 0.1, indicating that the detected OPEs in the surveyed area pose low risk to algae, crustaceans, and fish. These findings were in line with those found in environmental waters such as the Bohai Sea [24], Yellow Sea [37], Jiaozhou Bay [35], Beiyun and Yongding rivers [34], Taihu Lake and its inflowing rivers, and Beijing–Hangzhou Grand Canal [37]. However, the maximum RQs of several OPEs (i.e., TEP, TCEP, TPPO, EHDPP, TPhP) suggested moderate risks in surface water from Nansi Lake and Lunan Canal [27], Yarlung Tsangpo River and Lhasa River [22], and seawater from Xiangshan Bay [10]. Particularly, TEHP exhibited high risk in parts of the Pearl River Basin [43]. Therefore, the ecological risks of OPEs to aquatic organisms still deserve long-term attention.

Nevertheless, it is worth noting that the ecological risk assessment in this work is simplified, relying merely on an acute-toxicity deterministic RQ model with AF = 1000. This method ignores chronic risks and OPE mixture toxicity and may underestimate aquatic hazards; more rigorous approaches cover chronic toxicity, mixture toxicity, probabilistic risk assessment and species sensitivity distribution. Follow-up studies will gather comprehensive toxicological data to implement multi-dimensional advanced risk assessment.

## 3. Materials and Methods

### 3.1. Chemicals and Reagents

Nineteen standard OPEs (>98% purity), i.e., trimethyl phosphate (TEP), tri-isopropyl phosphate (TiPrP), tripropyl phosphate (TPrP), tri-isobutyl phosphate (TiBP), tributyl phosphate (TBP), tris(2-chloroethyl) phosphate (TCEP), tris(1-chloro-2-propyl) phosphate (TCIPP), tripentyl phosphate (TPeP), tris(3-chloropropyl) phosphate (TCPP), tris(1,3-dichloro-2-propyl) phosphate (TDCPP), triphenyl phosphate (TPhP), tris(2-butoxyethyl) phosphate (TBOEP), 2-ethylhexyl diphenyl phosphate (EHDPP), tris(2-ethylhexyl) phosphate (TEHP), triphenylphosphine oxide (TPPO), tri-o-cresyl phosphate (TOCP), tri-m-cresyl phosphate (TMCP), tri-p-cresyl phosphate (TPCP), and tris(2-isopropylphenyl) phosphate (TiPPP), were purchased from Alta Scientific (Tianjin, China). Their corresponding 9 ILISs, including TEP-D_15_, TPrP-D_21_, TBP-D_27_, TCEP-D_12_, TCIPP-D_18_, TDCPP-D_15_, TPhP-D_15_, TBOEP-D_27_, and TEHP-D_51_, were supplied by Alta Scientific (Tianjin, China). Stock standard solutions of 19 OPEs (1 mg/L) and 9 ILISs (1 mg/L) were prepared in isooctane and stored at −18 °C in the dark, remaining stable for three months. Daily calibration standard solutions were prepared by serial dilution of the stock solutions with isooctane.

Chromatographic-grade hexane, dichloromethane (DCM), ethyl acetate, acetone, and isooctane were obtained from Merck (Darmstadt, Germany), and anhydrous sodium sulfate was purchased from Sinopharm Chemical Reagent (Beijing, China). Ultrapure water (18.2 MΩ·cm, 25 °C) was prepared from Milli-Q Plus 185 (Millipore, Burlington, MA, USA).

### 3.2. Sampling and Preparation

A total of twenty-six water samples were collected in November and December of 2025. Three Wahaha pure water samples were purchased from local stores. Three tap water samples were collected from local families. Six river water samples were collected from an urban river in Zhoushan (R1–R6). Eight seawater samples were collected from Sanmen Bay (S1–S8), East China. The sampling locations for river water and seawater are shown in Figure S3. The collected water samples were stored at 0–4 °C and subjected to automated LLE as soon as possible, with the maximum storage period controlled within 7 days.

### 3.3. Automated Liquid–Liquid Extraction

Water samples were extracted with hexane and DCM. Briefly, 500 mL of water sample spiked with 50 ng of 9 ILISs was extracted with 25 mL of hexane and 25 mL of DCM by automated LLE for 10 min using an AG-LDZ-6 separating funnel oscillator (Shanghai Ou Ge Electronics Co. Ltd., Shanghai, China), respectively. After allowing for phase separation (5 min), the organic phases were successively decanted through anhydrous sodium sulfate and concentrated to dryness at 40 °C using a DryVap™ concentrator system (Horizon technology, Lake Forest, CA, USA). The residues were reconstituted in 200 μL of isooctane for GC-MS/MS analysis.

### 3.4. Instrumental Analysis

OPEs were analyzed on a 7890B/7000C Triple Quad GC-MS/MS in EI mode at 70 eV (Agilent Technologies, Santa Clara, CA, USA). In total, 1 μL was injected in splitless mode into a HP-5 MS capillary column (30 m × 0.25 mm × 0.25 μm) at an inlet temperature of 300 °C. Carrier gas was high-purity helium at a constant flow of 1 mL/min. The oven temperature was initially programmed at 40 °C for 2 min and then increased to 180 °C at 5 °C/min (held for 1 min), ramped up to 240 °C at 3 °C/min (held for 5 min), and finally increased to 310 °C at 10 °C/min (held for 5 min). The temperatures of the transfer line and ion source were maintained at 300 °C and 300 °C, respectively. Multiple reaction monitoring (MRM) mode was chosen for quantitation, and the retention time and MRM parameters for each compound are summarized in Table 3. A representative GC-EI-MS/MS (MRM) chromatogram is shown in Figure S2.

### 3.5. Quality Assurance and Quality Control

Given that OPEs are pervasive environmental contaminants, minimizing background contamination is essential for accurate quantification. Empirical evidence suggests that the predominant contamination sources originate from organic solvents and plasticware [1,30]. To minimize background contamination, plasticware was entirely avoided throughout the experiment. All labware was made of glass and underwent rigorous cleaning procedures prior to use: glassware was first soaked in 5% nitric acid overnight, rinsed thoroughly with ultrapure water, sequentially washed with acetone and dichloromethane, and finally baked at 450 °C. Anhydrous sodium sulfate was calcined at 550 °C for 2 h and then cooled in a desiccator before application. All pretreatment steps were completed in a clean fume hood to reduce interference from laboratory airborne OPE contaminants. To accurately quantify exogenous OPE contamination and the carryover effect, a set of solvent blanks (one in every ten samples), procedural blanks (one in every ten samples), field blanks (one in each batch sampling), and trip blanks (one in each batch sampling) was measured for each analytical batch to subtract the background values. Furthermore, for each batch of samples, ILISs for each sample, matrix spikes, and sample duplicates were analyzed for quality control. No target OPEs were detected above LOD in all blanks, indicating negligible background contamination and carryover during pretreatment and instrumental analysis.

## 4. Conclusions

A simple and rapid method based on plastic-free automated LLE-GC-MS/MS was developed and validated for simultaneous enrichment and quantification of 19 OPEs in drinking and environmental water samples. Thirteen OPEs were detected across four water matrices: Wahaha pure water, tap water, river water, and seawater. Notably, TCEP and TCIPP emerged as the predominant compounds among the detected OPEs, possibly reflecting extensive use of these OPEs in the study area. The ecological risk assessment based on acute toxicity data indicated that ΣOPEs posed a low risk to algae, crustaceans, and fish. However, these preliminary conclusions are derived from limited samples of drinking water and environmental water. To validate the generalizability of these findings, comprehensive sampling should be conducted across multiple sites and seasons throughout Zhejiang Province and other regions of China. Beyond these follow-up studies, this study may stimulate broader interest in applying multivariate statistical analyses for identifying OPE sources and discerning their composition. Additionally, it encourages in-depth exploration of their spatial heterogeneity, seasonal dynamics, and the driving forces associated with meteorological conditions and anthropogenic emissions. Furthermore, taking into account the high prevalence of OPEs both in drinking and environmental water samples, long-term exposure and bioaccumulation of these compounds in human and aquatic organisms should be further explored. In addition, although GC-MS/MS achieved effective quantification of target OPEs in the present work, high-resolution mass spectrometry (Orbitrap/QTOF) is suggested to cross-validate the analytical data and identify unknown OPE transformation products via accurate mass measurement. More attention should be paid to the environmental degradation and transformation behaviors of OPEs, and the ecological risks derived from their transformation byproducts deserve in-depth discussion in follow-up investigations.

## Figures and Tables

**Table 1:** Analytical characteristics of the proposed method.

Analyte	ILIS	Linear Range (μg/L)	*r* ^2^	Instrumental LOD (μg/L)	Instrumental LOQ (μg/L)	Method LOD ^a^ (ng/L)	Method LOQ ^b^ (ng/L)	Precision, RSD (%, *n* = 6)	
								Intra-Day	Inter-Day
TEP	TEP-D_15_	5–1000	0.9984	1.30	4.05	0.52	1.62	12.6	15.6
TiPrP	TPrP-D_21_	5–1000	0.9976	1.15	3.55	0.46	1.42	5.6	15.1
TPrP	TPrP-D_21_	5–1000	0.9972	1.63	4.88	0.65	1.95	8.3	11.6
TiBP	TBP-D_27_	5–1000	0.9955	1.43	4.25	0.57	1.70	7.5	17.5
TBP	TBP-D_27_	5–1000	0.9947	1.73	5.00	0.69	2.00	13.8	17.4
TCEP	TCEP-D_12_	5–1000	0.9971	3.23	10.7	1.29	4.28	12.0	14.6
TCIPP	TCIPP-D_18_	5–1000	0.9979	2.53	7.63	1.01	3.05	15.0	16.9
TPeP	TBP-D_27_	5–1000	0.9911	1.43	4.45	0.57	1.78	16.6	11.3
TCPP	TCIPP-D_18_	5–1000	0.9917	3.28	10.6	1.31	4.26	6.8	10.8
TDCPP	TDCPP-D_15_	5–1000	0.9931	3.53	11.8	1.41	4.71	8.6	14.9
TPhP	TPhP-D_15_	5–1000	0.9967	4.43	14.9	1.77	5.96	9.6	11.8
TBOEP	TBOEP-D_27_	5–1000	0.9951	5.80	19.6	2.32	7.83	8.4	14.7
EHDPP	TPhP-D_15_	5–1000	0.9986	2.88	9.50	1.15	3.80	12.9	16.6
TEHP	TEHP-D_51_	5–1000	0.9923	1.95	6.50	0.78	2.60	15.5	10.9
TPPO	TPhP-D_15_	5–1000	0.9918	4.35	14.5	1.74	5.80	17.8	19.9
TOCP	TPhP-D_15_	5–1000	0.9941	4.38	14.6	1.75	5.82	12.2	15.1
TMCP	TPhP-D_15_	5–1000	0.9905	4.78	16.0	1.91	6.42	15.0	15.1
TPCP	TPhP-D_15_	5–1000	0.9909	4.55	15.1	1.82	6.05	16.8	12.5
TiPPP	TPhP-D_15_	5–1000	0.9943	3.93	13.0	1.57	5.20	16.0	15.6

^a^ Method LODs and ^b^ method LOQs are based on sample pretreatment (500 mL sample and 0.2 mL final volume).

**Table 2:** The comparison of the proposed method with other methods for OPE detection in water samples.

Method	N *	Sample Type	Sample Volume (mL)	Sorbent	Extract or Elution or Disperser Solvent	Extraction Processing Time (min)	Quantification Method	LOD (ng/L)	Recovery (%)	RSD (%)	Ref.
HS-SPME-GC-NPD	9	River water, pond water, tap water	10	Graphene oxide-based sol–gel stainless-steel fiber	/	~40 min	External standard method	1.4–135.6	80–112	<10	[12]
DIA-LC-MS/MS	12	Drinking water, surface water, ground water and wastewater	0.8	/	/	/	Isotope dilution (8 ILISs)	1.5–30	16–111	<14	[25]
dSPE-GC-MS	2	Seawater and wastewater	50	Hyper-crosslinked β-cyclodextrin polymer	5 mL of cyclohexane	~30 min	External standard method	57–141	53–98	≤13	[15]
Automated SPE-GC-MS/MS	13	Source water, finished water, terminal tap water	500	Oasis HLB cartridge	10 mL of ethyl acetate	~40 min	External standard method	0.4–12	32–131	≤17	[18]
SPE-GC-MS/MS	13	Seawater	1000	Oasis HLB CNW C18 tandem cartridges	20 mL of DCM	>200 min	External standard method	0.03–0.25	79–123	<54	[19]
SPE-GC-MS/MS	5	Ultrapure water, tap water, seawater, surface water, secondary effluent and swimming pool water	500	Oasis HLB cartridge (500 mg, 6 mL)	5 mL of methanol and 5 mL of methyl tert-butylether	>130 min	Isotope dilution (5 ILISs)	0.3–24	85–135	<22	[20]
SPE-GC-MS	10	Bottled drinking water	500	Oasis HLB cartridge (500 mg, 6 mL)	18 mL of acetonitrile	>100 min	Isotope dilution (3 ILISs)	0.3–12.3	71–97	<14	[1]
SPE UPLC-MS/MS	12	Lake water	1000	ENVI-18 solid-phase extraction column	8 mL of acetonitrile: DCM (3:1, *v*:*v*)	>343 min	Isotope dilution (3 ILISs)	0.01–1.72	83–117	/	[31]
Automated LLE-GC-MS	10	Tap water, purified water, and bottled water	600	/	60 mL of DCM twice	~60 min	External standard method	0.68–2.96	67–125	<29	[21]
Automated LLE-UPLC-MS/MS	9	Source water, before and after treatment plant water, and domestic tap water	50	/	30 mL of DCM twice	~60 min	Isotope dilution (4 ILISs)	0.06–1.57	74–92	<10	[29]
automated LLE-GC-MS/MS	19	Wahaha pure water, tap water, river water, seawater	500	/	25 mL of DCM and 25 mL of hexane	~20 min	Isotope dilution (9 ILISs)	0.40–2.32	70–120	≤18	Present work

* Number of OPEs evaluated.

**Table 3:** MRM parameters of OPEs.

	Analyte	Retention Time (min)	Precursor Ion (*m*/*z*) → Product Ion (*m*/*z*)	Collision Energy (eV)
1	TEP	14.491	155 → 99, 99 → 81 *	7, 20
2	TiPrP	16.478	125 → 99, 99 → 81 *	13, 24
3	TPrP	21.583	141 → 99, 99 → 81 *	3, 21
4	TiBP	24.899	155 → 99, 99 → 81 *	3, 22
5	TBP	27.946	155 → 99, 99 → 81 *	3, 25
6	TCEP	30.452	205 → 143, 143 → 117 *	7, 10
7	TCIPP	31.328	125 → 99, 99 → 81 *	10, 22
8	TPeP	34.506	169 → 99, 99 → 81 *	3, 23
9	TCPP	38.923	125 → 99, 99 → 81 *	12, 20
10	TDCPP	45.096	191 → 75, 99 → 81 *	15, 23
11	TPhP	46.685	326 → 215, 325 → 169 *	33, 28
12	TBOEP	47.334	153 → 125, 125 → 99 *	5, 12
13	EHDPP	47.556	251 → 77 *, 94 → 65	23, 28
14	TEHP	48.821	113 → 95, 99 → 81 *	16, 23
15	TPPO	49.716	201 → 77, 199 → 152 *	23, 32
16	TOCP	51.409	277 → 179 *, 165 → 115	17, 45
17	TMCP	53.194	368 → 165 *, 165 → 115	22, 40
18	TPCP	56.043	368 → 165 *, 165 → 164	30, 35
19	TiPPP	57.200	335 → 251, 118 → 91 *	11, 25
①	TEP-D_15_	14.235	167 → 103, 103 → 83 *	7, 20
②	TPrP-D_21_	21.262	199 → 103, 103 → 83 *	4, 22
③	TBP-D_27_	27.563	167 → 103, 103 → 83 *	4, 22
④	TCEP-D_12_	30.217	261 → 131, 148 → 120 *	10, 10
⑤	TCIPP-D_18_	30.988	103 → 83 *, 131 → 103	20, 12
⑥	TDCPP-D_15_	44.689	197 → 79, 103 → 83 *	12, 22
⑦	TPhP-D_15_	46.486	341 → 243, 339 → 178 *	16, 27
⑧	TBOEP-D_27_	46.853	126 → 100 *, 154 → 126	12, 5
⑨	TEHP-D_51_	47.962	117 → 85, 103 → 83 *	8, 22

* Quantitative ion.

## Data Availability

The original contributions presented in this study are included in the article and Supplementary Materials. Further inquiries can be directed to the corresponding authors.

## Supplementary Materials

The following supporting information can be downloaded at: https://www.mdpi.com/article/10.3390/molecules31122131/s1. References [22,44,45,46] are cited in the Supplementary Materials.
