# Supplementary material for: Determination and Ecological Risk Assessment of Organophosphate Esters in Drinking and Environmental Waters by Automated Liquid–Liquid Extraction Coupled with GC-MS/MS
Source: Molecules. 2026 Jun 17;31(12):2131. doi: 10.3390/molecules31122131 (PMC13304668; doi:10.3390/molecules31122131)
Supplement: Supplementary file 1 [file molecules-31-02131-s001.zip › molecules-4346183-supplementary.pdf]

SUPPORTING INFORMATION

Determination and Ecological Risk Assessment of Organophosphate Esters in Drinking and Environmental Waters by Automated Liquid–Liquid Extraction Coupled with GC-MS/MS

Guowei Wang [1,2], Hongmei Hu[1,2,*], Yanjian Jin[3,*], Tiejun Li[1,2], Zhenhua Li[1,2], Yunyong She[3], Qinglin Mu[3] and Yuanming Guo[1,2]

[1] Key Laboratory of Sustainable Utilization of Technology Research for Fisheries Resources of Zhejiang Province, Zhejiang Marine Fisheries Research Institute, Zhoushan 316021, China

2. Institute of Marine and Fisheries, Zhejiang Ocean University, Zhoushan 316021, China

[3] Marine Ecological and Environmental Monitoring Center of Zhejiang Province, Zhoushan 316021, China.

Corresponding Author

Hongmei Hu

*Phone: +86-580-2299883; Fax: +86-580-2299881; E-mail: huhm@zjou.edu.cn

Yanjian Jin

*Phone: +86-580-8263075; Fax: +86-580-2025873; E-mail: jinyanjian@126.com

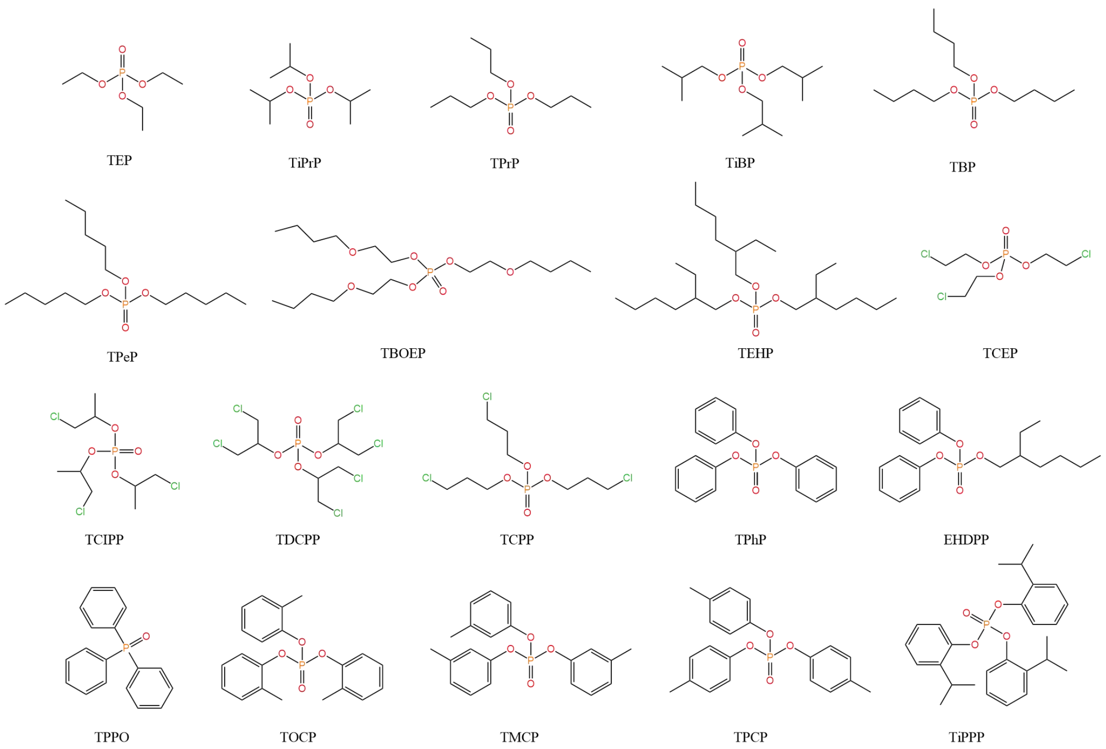

Figure S1. Chemical structures of the 19 OPEs.

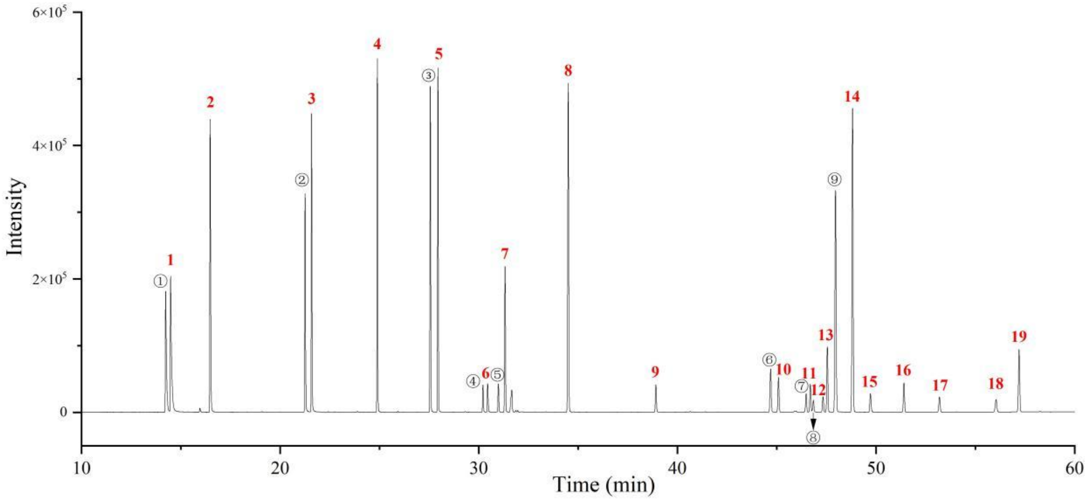

Figure S2. Chromatogram of 19 OPEs and 9 ILISs mixed standards by GC-MS/MS.

Peaks 1. TEP; 2. TiPrP; 3. TPrP; 4. TiBP; 5. TBP; 6. TCEP; 7. TCIPP; 8. TPeP; 9. TCPP; 10. TDCPP; 11. TPhP; 12. TBOEP; 13. EHDPP; 14. TEHP; 15. TPPO; 16. TOCP; 17. TMCP; 18. TPCP; 19. TiPPP; ①.TEP-D_{15}; ②.TPrP-D_{21}; ③.TBP-D_{27}; ④.TCEP-D_{12}; ⑤.TCIPP-D_{18}; ⑥.TDCPP-D_{15}; ⑦.TPhP-D_{15}; ⑧.TBOEP-D_{27}; ⑨.TEHP-D_{51}.

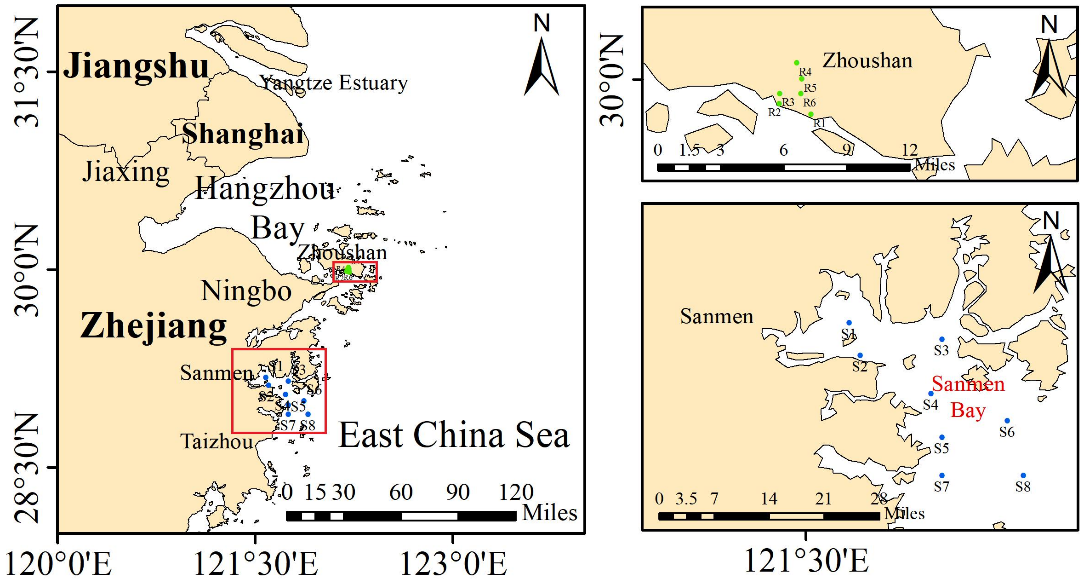

Figure S3. Map of sampling locations for river water of Zhoushan (R1–R6) and seawater of Sanmen Bay (S1–S8), East China.

Table S1. The physicochemical properties of 19 OPEs.

Abbreviation	Full Name	CAS No.	Chemical formula	Molecular weight	Log K_{OW}	Water solubility (mg/L)	Vapor Pressure (mm Hg)
TEP	triethyl phosphate	78-40-0	$C_6H_{15}O_4P$	182.15	0.80	5.0×10^{5}	3.9×10^{-1}
TiPrP	triisopropyl phosphate	513-02-0	$C_9H_{21}O_4P$	224.23	2.12	5.01×10^{2}	0.14
TPrP	tripropyl phosphate	513-08-6	$C_9H_{21}O_4P$	224.23	1.87	827	2.9×10^{-2}
TiBP	triisobutyl phosphate	126-71-6	$C_{12}H_{27}O_4P$	266.32	3.60	16.2	1.28×10^{-2}
TBP	tributyl phosphate	126-73-8	$C_{12}H_{27}O_4P$	266.31	4.00	280	1.13×10^{-3}
TPeP	tripentyl Phosphate	2528-38-3	$C_{15}H_{33}O_4P$	308.39	5.29	0.33	1.67×10^{-5}
TBOEP	tris(2-butoxyethyl) phosphate	78-51-3	$C_{18}H_{39}O_7P$	398.47	3.75	1.2×10^{3}	1.23×10^{-6}
TEHP	tris(2-ethylhexyl) phosphate	78-42-2	$C_{24}H_{51}O_4P$	434.63	9.49	0.6	2.0×10^{-6}
TCEP	tris(2-chloroethyl) phosphate	115-96-8	$C_6H_{12}Cl_3O_4P$	258.49	1.44	7.4×10^{3}	1.1×10^{-4}
TCIPP	tris(1-chloro-2-propyl) phosphate	13674-84-5	$C_9H_{18}Cl_3O_4P$	327.57	2.59	1.6×10^{3}	1.9×10^{-6}
TDCPP	tris(1,3-dichloro-2-propyl) phosphate	13674-87-8	$C_9H_{15}Cl_6O_4P$	430.89	3.85	1.5	7.4×10^{-8}
TCPP	tris(3-chloropropyl) phosphate	1067-98-7	$C_9H_{18}Cl_3O_4P$	327.57	3.11	18.8	4.79×10^{-6}
TPhP	triphenyl phosphate	115-86-6	$C_{18}H_{15}O_4P$	326.29	2.88	82.38	9.1×10^{-8}
EHDPP	2-ethylhexyl diphenyl phosphate	1241-94-7	$C_{20}H_{27}O_4P$	326.40	5.73	1.9	5.0×10^{-5}
TPPO	triphenylphosphine oxide	791-28-6	$C_{18}H_{15}OP$	278.28	2.87	62.8	2.6×10^{-9}
TOCP	tri-o-cresyl phosphate	78-30-8	$C_{21}H_{21}O_4P$	368.36	5.11	0.36	6.00×10^{-7}
TMCP	tri-m-cresyl phosphate	563-04-2	$C_{21}H_{21}O_4P$	368.36	6.34	1.84×10^{-2}	1.09×10^{-7}
TPCP	tri-p-cresyl phosphate	78-32-0	$C_{21}H_{21}O_4P$	368.36	6.34	0.30	3.49×10^{-8}
TiPPP	tris(2-isopropylphenyl) phosphate	64532-95-2	$C_{30}H_{39}O_4P$	452.52	9.07	2.59×10^{-5}	2.06×10^{-8}

Table S2. Recoveries of 19 target OPEs at four spiking levels in wahaha pure water, tap water, river water, and seawater by the proposed automated LLE GC-MS/MS method.

Analyte	Wahaha pure water (n=6)		Tap water (n=6)		River water (n=6)		Seawater (n=6)	
	Spiked: 20 ng/L		Spiked: 50 ng/L		Spiked:100 ng/L		Spiked: 400 ng/L	
	Recovery (%)	RSD (%)	Recovery (%)	RSD (%)	Recovery (%)	RSD (%)	Recovery (%)	RSD (%)
TEP	78	12.3	83	16.7	77	15.9	90	1.3
TiPrP	71	11.2	74	10.8	78	10.0	76	5.8
TPrP	77	14.3	83	11.9	83	11.1	76	2.9
TiBP	80	16.6	95	13.5	71	12.9	90	10.4
TBP	77	14.8	81	16.8	75	15.8	109	6.0
TCEP	73	12.7	79	14.2	79	13.7	82	2.2
TCIPP	96	13.3	83	15.0	71	10.7	83	1.2
TPeP	79	15.8	80	18.0	75	12.6	93	6.1
TCPP	85	17.7	71	17.1	85	15.0	87	17.7
TDCPP	76	15.3	73	13.0	71	12.7	107	7.5
TPhP	71	6.4	72	6.9	70	12.0	95	4.2
TBOEP	77	7.5	70	6.5	87	16.9	112	11.2
EHDPP	92	14.5	84	17.8	83	15.7	120	8.9
TEHP	85	8.6	74	9.1	70	5.8	106	10.9
TPPO	83	13.2	76	18.0	84	15.1	70	8.2
TOCP	76	15.6	82	16.3	79	16.4	93	5.7
TMCP	84	14.6	81	16.4	83	16.2	105	6.6
TPCP	87	16.0	79	15.6	81	15.8	116	5.8
TiPPP	80	15.4	95	12.9	87	12.2	85	5.0

Table S3. Concentrations (ng/L) of detected OPEs in Wahaha pure water, tap water, river water of Zhoushan and seawater of Daiquyang and Yueqing Bay.

		TEP	TiPrP	TPrP	TiBP	TBP	TCEP	TCIPP	TDCPP	TPhP	TBOEP	EHDPP	TEHP	TPPO	∑OPEs
Wahaha pure water (n=3)	Mean	ND	ND	ND	ND	ND	8.60	3.11	ND	ND	ND	ND	ND	1.21	12.9
	Min	ND	ND	ND	ND	ND	4.92	2.01	ND	ND	ND	ND	ND	ND	8.23
	Max	ND	ND	ND	ND	ND	12.7	4.89	ND	ND	ND	ND	ND	1.88	18.5
	DF(%)	0	0	0	0	0	100	100	0	0	0	0	0	33.3	100
Tap water (n=3)	Mean	31.3	2.12	1.02	9.33	2.14	68.5	36.9	6.09	ND	ND	ND	ND	31.3	189
	Min	26.4	1.80	0.79	8.41	1.77	52.4	32.8	5.83	ND	ND	ND	ND	28.6	159
	Max	39.8	2.29	1.22	10.0	2.62	89.4	44.4	6.41	ND	ND	ND	ND	34.7	218
	DF(%)	100	100	100	100	100	100	100	100	0	0	0	0	100	100
River water of Zhoushan (n=6)	Mean	34.2	6.28	4.77	22.0	13.1	122	67.2	9.01	ND	2.11	ND	ND	105	386
	Min	22.9	2.38	1.33	11.0	11.0	61.4	39.1	4.93	ND	ND	ND	ND	36.0	202
	Max	43.9	9.32	9.02	33.2	19.5	288	150	14.7	ND	5.16	ND	ND	267	623
	DF(%)	100	100	100	100	100	100	100	100	0	33.3	0	0	100	100
Seawater of Sanmen Bay (n=8)	Mean	19.1	ND	ND	9.90	8.17	31.4	30.5	0.96	1.09	5.64	1.58	0.54	31.0	140
	Min	14.8	ND	ND	3.33	2.59	16.2	17.5	ND	ND	ND	ND	ND	21.2	111
	Max	23.9	ND	ND	18.7	16.8	54.4	62.5	1.95	2.50	14.3	7.36	1.59	44.5	175
	DF(%)	100	0	0	100	100	100	100	25	12.5	50	37.5	12.5	100	100

DF, detection frequency.

ND, not detected.

Table S4. Toxicity data and PNECs of detected OPEs on three different trophic levels aquatic organisms.

Analytes	Class	Species	Toxicity data (EC_{50}/LC_{50}, mg/L)	PNEC (ng/L)	References
TEP	Algae	*Scenedesmus subspicatus*	900	900000	[44]
	Crustaceans	*Daphnia magna*	350	350000	[44]
	Fish	*Lepomis macrochirus*	2140	2140000	[44]
TPrP	Algae	/	/	/	/
	Crustaceans	/	/	/	/
	Fish	*Danio rerio*	252	252000	[45]
TiBP	Algae	*Scenedesmus subspicatus*	34	34000	[44]
	Crustaceans	*Daphnia magna*	11	11000	[44]
	Fish	*Leuciscus idus*	20	20000	[44]
TBP	Algae	*Scenedesmus subspicatus*	4.2	4200	[44]
	Crustaceans	*Daphnia magna*	3.65	3650	[44]
	Fish	*Carassius auratus*	8.8	8800	[44]
TCEP	Algae	*Scenedesmus subspicatus*	51	51000	[44]
	Crustaceans	*Daphnia magna*	330	330000	[44]
	Fish	*Carassius auratus*	90	90000	[44]
TCIPP	Algae	*Scenedesmus subspicatus*	45	45000	[44]
	Crustaceans	*Daphnia magna*	91	91000	[44]
	Fish	*Poecilia reticulata*	30	30000	[44]
TDCPP	Algae	*Pseudokirchneriella subcapitata*	39	39000	[44]
	Crustaceans	*Daphnia magna*	4.2	4200	[44]
	Fish	*Carassius auratus*	5.1	5100	[44]
TPhP	Algae	*Scenedesmus subspicatus*	0.5	500	[44]
	Crustaceans	*Daphnia magna*	1	1000	[44]
	Fish	*Carassius auratus*	0.7	700	[44]
TBOEP	Algae	/	/	/	/
	Crustaceans	*Daphnia magna*	75	75000	[44]
	Fish	*Pimephales promelas*	13	13000	[44]
EHDPP	Algae	/	/	/	/
	Crustaceans	*Daphnia magna*	0.31	310	[46]
	Fish	/	/	/	/
TEHP	Algae	/	/	/	/
	Crustaceans	*Daphnia magna*	0.74	740	[46]
	Fish	*Oryzias latipes*	500	500000	[46]
TPPO	Algae	NA	20.4	20400	[22]
	Crustaceans	*Daphnia magna*	42.7	42700	[22]
	Fish	NA	46	46000	[22]

/: The data was not available.
NA: not available.
